# Investigating the Association Between the Co-Occurrence of Behavioral Health Risk Factors and Sick Days in General Hospital Patients

**DOI:** 10.3389/ijph.2022.1605215

**Published:** 2022-09-27

**Authors:** Marie Spielmann, Anika Tiede, Filipa Krolo, Kornelia Sadewasser, Ali Alexander Aghdassi, Chia-Jung Busch, Peter Hinz, Julia van der Linde, Ulrich John, Jennis Freyer-Adam

**Affiliations:** ^1^ Institute for Medical Psychology, University Medicine Greifswald, Greifswald, Germany; ^2^ German Centre for Cardiovascular Research, Partner Site Greifswald, Greifswald, Germany; ^3^ Department of Internal Medicine A, University Medicine Greifswald, Greifswald, Germany; ^4^ Department of Otorhinolaryngology, University Medicine Greifswald, Greifswald, Germany; ^5^ Department of Trauma, Reconstructive Surgery and Rehabilitation Medicine, University Medicine Greifswald, Greifswald, Germany; ^6^ Department of General, Visceral, and Thoracic Surgery, University Medicine Greifswald, Greifswald, Germany; ^7^ Department of Prevention Research and Social Medicine, University Medicine Greifswald, Greifswald, Germany

**Keywords:** health behaviors, health risk factors, co-occurrence, accumulation, sick days

## Abstract

**Objectives:** To investigate the co-occurrence of 4 behavioral health risk factors (BHRFs), namely tobacco smoking, alcohol at-risk drinking, physical inactivity and unhealthy diet and their association with sick days prior to hospitalization in general hospital patients.

**Methods:** Over 10 weeks (11/2020-04/2021), all 18-64-year-old patients admitted to internal medicine, general and trauma surgery, and otorhinolaryngology wards of a tertiary care hospital were systematically approached. Among 355 eligible patients, 278 (78.3%) participated, and 256 (72.1%) were analyzed. Three BHRF sum scores were determined, including current tobacco smoking, alcohol use, physical inactivity and 1 of 3 indicators of unhealthy diet. Associations between BHRF sum scores and sick days in the past 6 months were analyzed using multivariate zero-inflated negative binomial regressions.

**Results:** Sixty-two percent reported multiple BHRFs (≥2). The BHRF sum score was related to the number of sick days if any (*p* = 0.009) with insufficient vegetable and fruit intake as diet indicator.

**Conclusion:** The majority of patients disclosed multiple BHRFs. These were associated with sick days prior to admission. The findings support the need to implement interventions targeting multiple BHRFs in general hospitals.

## Introduction

Behavioral health risk factors (BHRFs) like tobacco smoking, alcohol at-risk drinking, unhealthy diet and physical inactivity are main preventable causes of non-communicable diseases in western countries [1]. The risk of mortality increases with the number of BHRFs [2, 3]. In addition, the coexistence of BHRFs might not only have additive, but multiplicative effects; e.g. on the risk of esophageal or head and neck cancer [4, 5].

More than half of the adult population in western countries have ≥2 of the 4 BHRFs, when using overweight as an indicator of unhealthy diet; and numbers are even higher when insufficient vegetable and fruit intake is used as an indicator of unhealthy diet instead or in addition to overweight [6–9]. The co-occurrence of BHRFs appears to be particularly prevalent among general hospital patients. While in Germany 56% of the general population aged 18+ years reported ≥2 of the 4 BHRFs, 66% of German general hospital patients aged 18–64 years did so [7, 10]. However, as overweight is often used as diet indicator, non-overweight people who practice unhealthy diet may remain undetected.

One main problem in this respect is the need for a time-saving assessment of unhealthy diet. As the assessment of unhealthy diet is complex, 3 indicators of it might be considered: overweight, low vegetable and fruit intake, and the co-occurrence of low fiber and high sugar, fat and salt intake. Overweight and low vegetable and fruit intake have been used in several studies [6, 7, 11]. The limitation of such simple measures is that they do not sufficiently cover important aspects of a healthy diet. Fifteen dietary components, including unhealthy fat-, fiber-, salt- and sugar intake, are part of the leading risks responsible for global burden of disease [1] and a diet rich in fiber and low in sugar, fat and salt is recommended to prevent non-communicable chronic diseases [12]. Little is known about the co-occurrence of low fiber and high sugar, fat and salt intake.

There is clear evidence that BHRFs are related to morbidity and mortality in a dose-dependent manner [13–16]. However, there is a lack of knowledge about other indicators of ill health such as sick leave at the work place and how they relate to the co-occurrence of BHRFs. As single BHRFs are associated with sick leave due to health disorder [17], it could be assumed that the number of co-occurring BHRFs might correlate with the number of sick days.

The aims of this study are to examine the co-occurrence of BHRFs among general hospital patients and to explore the association between BHRFs and the number of self-reported sick days.

## Methods

### Sampling Frame and Participants

Data from a survey conducted as part of the research project “Proactive automatized lifestyle-intervention for cancer prevention, PAL” were analyzed.

Over 10 weeks participants were recruited in 4 major medical departments of a University Hospital in Germany: internal medicine (i.e., gastroenterology, angiology, pneumology, endocrinology and nephrology), general surgery, trauma surgery and otorhinolaryngology. Due to Covid-19-related restrictions, recruitment was conducted over 2 phases (2020/11/03 to 2020/12/11; 2021/03/09 to 2021/04/01) as during Covid-19 waves 2 and 3, hospital admissions were limited to only highly urgent cases; and the participation of cardiology wards was not possible due to the treatment of Covid-19 patients.

On Tuesdays through Fridays, all patients aged 18–64 years, admitted to the participating wards on the previous day were approached. Patients were asked to fill in a questionnaire assessing health risk behaviors and health-status using tablet computers. Patients cognitively or physically incapable or terminally ill (*n* = 9), with highly infectious diseases (*n* = 21), discharged or transferred outside the study area within the first 24 h (*n* = 43), already recruited for the study during an earlier hospital stay (*n* = 22), with insufficient language skills (*n* = 11), or employed at the conducting research institute or hospitalized relatives (*n* = 0) were excluded.

Of 461 admitted patients, 355 met inclusion criteria and were eligible. Among them, 42 declined participation, 31 were missed or interrupted in completing the survey due to discharge or transferral; 4 were not reached over 3 recruitment days, resulting in 278 respondents (78.3% of eligible). Due to technical problems, data from 22 participants were lost, leaving 256 participants for the final analysis.

### Measurements

All assessments are based on self-report.

#### BHRFs

Tobacco smoking was assessed using the question “Do you currently smoke?” with 4 response-categories: current daily smoking, occasional smoking, former smoking and never smoking. Current smoking was considered as BHRF. Number of cigarettes per day was assessed for those reporting daily smoking by the question: “How many cigarettes did you usually smoke per day during the last 4 weeks prior to hospital admission?”.

Alcohol at-risk drinking was determined using the Alcohol Use Disorder Identification Test-Consumption (AUDIT-C [18]), with a score range 0–12. The AUDIT-C consists of 3 items on frequency and quantity of drinking and on frequency of drinking 4 or more glasses per occasion among females and 5 or more among males (heavy drinking). Alcohol at-risk drinking was defined for AUDIT-C-score of ≥4 for females and ≥5 for males [19].

Unhealthy diet was measured using 3 indicators: overweight, insufficient vegetable and fruit intake, and a combined measure of unhealthy intake of fat, fiber, salt, and sugar. Overweight was assessed using the body-mass-index obtained by self-reported weight in kilograms and height in meters. A body-mass-index ≥25 was defined as diet risk factor overweight [20]. Insufficient vegetable and fruit intake was derived from the question “How many servings of vegetables and fruits do you eat per day on average?”. Less than 5 servings were considered as diet risk factor insufficient vegetable and fruit intake [12]. A combined measure of unhealthy intake of fat, fiber, salt and sugar was used. As to our knowledge, no brief measure exists that assesses those 4 nutrients, 16 questions on the number of servings consumed of different foods per day or per week similar to the Mediterranean Diet Adherence Screener [21] were developed. Each item was supported by examples of what 1 serving could include, based on mean nutritional values determined by a nutritional scientist. Fat intake was assessed by 9 items (cheese, instant meals, salted snacks, eggs, fatty fish, red meat, processed meat, butter/oil, milk), fiber by 4 items (vegetable and fruit, other food rich in fiber, bread), salt by 6 items (cheese, instant meals, salted snacks, red meat, processed meat, bread), and sugar by 3 items (sweets, sugar/honey, sweetened drinks). To identify whether the diet risk factors fat, fiber, salt and sugar were present, current recommendations were used, resulting in cut-off values of ≥30% of total energy intake from fat [22], <30 g of fiber per day [12], ≥5 g of salt per day [23] and ≥50 g of sugar per day [24]. Daily fat intake in gram was converted into kilocalories (kcal). Recommended fat intake per day depends on the basal metabolism and on the level of physical activity. The basal metabolism was calculated for each participant according to the Harris-Benedict equation [25]. That is for women: 655.1+(9.6*weight in kg)+(1.8*height in cm)–(4.7*age in years) and for men: 66.47+(13.7*weight in kg)+(5*height in cm)–(6.8*age in years). To calculate the daily energy expenditure, the result was multiplied by a factor describing the physical activity level. Participants reporting no physical activity except for walking or sitting as part of the below reported measurement received the factor 1.2. Those with <150 min/150–1200 min/1200–2400 min/>2400 min of physical activity per week received 1.4/1.6/1.8/2.0, respectively. Assuming that participants’ total calorie intake corresponds to their daily energy expenditure, those with a daily fat-intake in kcal ≥0.3*(daily energy expenditure) were considered to consume too much fat. If recommendations for 1 or more of the 4 components were not met, participants were considered to have the diet risk factor unhealthy intake of fat, fiber, salt and sugar.

Physical inactivity was assessed using the International Physical Activity Questionnaire-Short form (IPAQ-Short [26–29]). It consists of 7 items, asking for the number of days per week of moderate and vigorous physical activity and for the minutes of physical activity on these days. While the IPAQ-Short typically assesses this information for the past week, this study asked for a typical week as the participants may not have been well enough for their usual level of physical activity the week prior to hospital admission. Based on the frequency and quantity information, the number of moderate physically active minutes per week was calculated. Minutes of vigorous activity were multiplied by 2 to convert them into minutes of moderate physical activity and then added to the minutes of reported moderate activity. In line with recommendations of the World Health Organization (WHO [30, 31]), reporting less than 150 min of moderate physical activity per week was considered as BHRF physical inactivity.

Three alternative BHRF sum scores were calculated for each participant. Each of the 4 BHRFs was counted as 1 when present and 0 when absent. This way, BHRF sum scores ranged from 0 to 4. Each sum score included a different indicator for unhealthy diet.

#### Outcome

The number of sick days was assessed by the question “On how many days in the past 6 months, did you receive a sick certificate or were you unable to do your task (e.g., work, educational training, job-seeking, household/gardening chores) for health reasons?” [32].

#### Descriptive Variables and Co-Variates

Sex (male, female) and age in years were assessed. School education was categorized into low (i.e., <10 years of school), medium (10–11 years) and high level of school education (>11 years).

The presence of non-communicable diseases, i.e., cancer diseases, chronic cardiovascular diseases, chronic respiratory diseases and diabetes mellitus were recorded by the question “Have you ever been diagnosed with … ?” with 5 response categories: No, and there is no such suspicion/No, but there is a suspicion at the moment/Yes, first during my current hospital stay/Yes, during the past year/Yes, more than 1 year ago/I don’t know. “Yes”-responses were regarded as “disease present”. All present NCDs were added up resulting in an NCD number ranging between 0 and 4. Self-rated health was assessed by asking “In general, how would you rate your current health status?” with the response categories “excellent” [1], “very good” [2], “good” [3], “fair” [4] and “poor” [5, 33].

### Statistical Analyses

Proportions and 95% confidence intervals are given for each of the 4 single BHRFs, for each of the 3 BHRF sum scores (0-4) and for the occurrence of ≥1 BHRFs and ≥2 BHRFs. Mean value and standard deviation were calculated for each BHRF and for the 3 BHRF sum scores. Due to the high number of zero values on sick days, we used zero-inflated negative binomial regression to investigate the association between BHRF sum scores and sick days. The binary model part describes the association with reporting any sick days, while the count model part describes the association with the number of sick days if any reported. Three zero-inflated negative binomial regression analyses with the 3 different BHRF sum scores as predictors and controlled for sex, age, school education and the number of NCDs were performed. Odds ratios (ORs) are given for the effect of BHRF sum scores on reporting any sick days (binary model part) and incidence rate ratios (IRRs) are given for the effect on the number of sick days if any reported (count model part). Due to multiple testing, Bonferroni’s correction was used resulting in *p*-values <0.017 being considered as statistically significant. One participant with missing values on the AUDIT-C was excluded from any analysis including alcohol measures and 1 with missing information on school education was excluded from all regression analyses. For the data analysis, STATA version 14.2 SE was used.

## Results

### Sample Characteristics

The mean age of the patient cohort was 49.4 years (SD = 12.3) and 56.3% were male. As depicted in Table 1, 17.7% had the lowest level of school education, and 46.1% had at least 1 non-communicable disease. The number of NCDs was on average 0.7 (SD = 0.9). Of all participants, 18.8% had cancer, 22.3% a cardiovascular disease, 16.0% a chronic respiratory disease and 12.9% diabetes mellitus. Self-rated health was on average 3.3 (SD = 0.9) and the mean number of sick days in the past 6 months was 28.1 (SD = 55.9).

**TABLE 1:** Sample characteristics (*n* = 256), Proactive automatized lifestyle-intervention for cancer prevention, Germany, 2020-2021.

Variables	*n*	%
Sex		
Male	144	56.3
Female	112	43.7
School education		
Low	45	17.7
Medium	160	62.8
High	50	19.6
Any non-communicable disease	118	46.1
Cancer disease	47	18.4
Cardiovascular disease	57	22.3
Chronic respiratory disease	43	16.8
Diabetes mellitus	33	12.9
	M	SD
Age	49.4	12.3
Number of non-communicable diseases	0.7	0.9
Self-rated health	3.3	0.9
Number of sick days in past 6 months	28.1	55.9

Notes: *n*, number of participants; M, mean; SD, standard deviation.

### Occurrence and Co-Occurrence of BHRFs

The most prevalent BHRF was unhealthy diet, regardless of whether overweight (61.7%), insufficient vegetable and fruit intake (86.7%) or the combined measure of unhealthy intake of fat, fiber, salt and sugar (100.0%) were used as indicator (Table 2). The second most frequent BHRF was tobacco smoking (39.1%), followed by physical inactivity (34.0%) and at-risk alcohol use (25.5%).

**TABLE 2:** Occurrence of behavioral health risk factors, Proactive automatized lifestyle-intervention for cancer prevention, Germany, 2020-2021.

Behavioral health risk factors	*n*	Mean	SD	At-risk		
				*n*	%	95% CI
Tobacco smoking						
Number of cigarettes per day	92^a^	15.0	11.4	100^b^	39.1	3.0–45.3
Alcohol use						
AUDIT-C Score	255	2.7	2.5	65	25.5	20.3–31.3
Physical inactivity						
Minutes physically active per week	256	765.7	1106.3	87	34.0	59.9–71.8
Unhealthy diet						
Indicator overweight						
Body mass index	256	27.6	6.1	158	61.7	55.5–67.7
Indicator vegetable/fruit						
Number of servings per day	256	2.6	2.0	222	86.7	81.9–90.6
Indicator fat/fiber/salt/sugar (FFSS)						
Number of FFSS risk factors^c^ ^,^ ^d^	256	2.4	1.1	256	100.0	98.6–100.0^e^
Fat in kilo calories per day	256	1086.8	624.2	167	65.2	59.1–71.1
Fiber in gram per day	256	18.7	10.0	231	90.2	85.9–93.6
Salt in gram per day	256	5.6	3.2	123	48.1	41.8–54.4
Sugar in gram per day	256	64.2	65.7	114	44.5	38.3–50.8

a current daily smokers.

b current daily and occasional smokers.

c of the 4 diet risk factors fat, fiber, salt, sugar.

d either diet risk factor fat, fiber, salt or sugar present.

e one-sided (97.5% confidence interval); *n*, number of participants; SD, standard deviation; CI, confidence interval.

The proportions of participants with ≥1 of the 4 BHRFs were 87.9% and higher, depending on whether overweight (87.9%), insufficient vegetable and fruit intake (93.7%) or the combined measure of fat, fiber, salt and sugar intake (100.0%) were used as indicators of unhealthy diet (Table 3).

**TABLE 3:** Co-occurrence of tobacco smoking, at-risk alcohol use, physical inactivity and unhealthy diet measured by 3 sum scores that either use overweight, intake of vegetable/fruit or intake of fat/fiber/salt/sugar as indicator for unhealthy diet (*n*, %, 95% CI), Proactive automatized lifestyle-intervention for cancer prevention, Germany, 2020-2021.

BHRF sum score	BHRF sum score containing unhealthy diet indicator								
	Overweight			Vegetable/fruit			Fat/Fiber/Salt/Sugar		
Average (*n*, M, SD)	255	1.6	1.0	255	1.9	1.0	255	2.0	0.9
0	31	12.2	(8.4-16.8)	16	6.3	(3.6–10.0)	0	0.0	(0–0.01) *
1	89	34.9	(29.1–41.1)	82	32.2	(26.5–38.3)	88	34.5	(28.7–40.7)
2	91	35.7	(29.8–41.9)	92	36.1	(30.2–42.3)	95	37.3	(31.3–43.5)
3	39	15.3	(11.1–20.3)	54	21.2	(16.3–26.7)	60	23.5	(18.5–29.2)
4	5	2.0	(0.6–4.5)	11	4.3	(2.2–7.6)	12	4.7	(2.5–8.1)
≥1	225	87.9	(83.3–91.6)	240	93.7	(90.0–96.4)	255	100.0	(98.6–100.0) *
≥2	136	53.1	(46.8–59.4)	158	61.5	(55.5–67.7)	168	65.6	(59.5–71.4)

Notes: *n*, number of participants; M, Mean; SD, standard deviation; CI, confidence interval; *one-sided (97.5% CI).

Likewise, the proportions of participants with ≥2 of the 4 BHRFs were 53.1% when using overweight, 61.5% when using insufficient vegetable and fruit intake and 65.6% when using the combined measure as diet indicator.

### Association Between BHRFs and Sick Days

Among all study participants, there was no association between BHRF sum score and reporting any sick days, regardless of the indicator, which was used to measure unhealthy diet (overweight: OR = 1.39, 95% CI: 0.88–2.20, *p* = 0.155; vegetable/fruit: OR = 1.38, 95% CI: 0.91–2.10, *p* = 0.130; fat/fiber/salt/sugar: OR = 1.41, 95% CI: 0.89–2.23, *p* = 0.140).

Among the study participants with sick days, a significant and positive association between BHRF sum score and the number of sick days was found when vegetable and fruit intake was used as indicator for unhealthy diet (IRR = 1.53, 95% CI: 1.11–2.09, *p* = 0.009). This association was not or only marginally significant when overweight (IRR = 1.27, 95% CI: 0.97–1.66, *p* = 0.086) and the combined measure of 4 food components (IRR = 1.46, 95% CI: 1.04–2.06, *p* = 0.031) were used as indicators of unhealthy diet as part of the BHRF-score calculation.

Among the covariates, NCD-number showed positive associations in each of the three count-models (*p*s = 0.012–0.016). Age, sex and school education were neither significantly associated with reporting any sick days in the binary model part nor with the number of sick days in the count model part.

## Discussion

Little is known about the association between co-occurring BHRFs and the number of sick days in hospitalized patients. The 3 key findings of this study are: 1) More than half of the general hospital patients reported multiple BHRFs. 2) The detailed assessment of unhealthy diet showed that unhealthy diet was the most prevalent BHRF regardless of which indicator was used. 3) The number of BHRFs was significantly related to the number of sick days in the 6 months prior to hospitalization when vegetable and fruit intake was used to measure unhealthy diet.

In contrast to a previous study showing unhealthy diet and physical inactivity to be the 2 most common BHRFs in hospital patients [10], our study revealed unhealthy diet followed by tobacco smoking to be most common. One reason might be the selection of medical departments in which the prevalence of BHRFs might differ. For example, cardiology, neurology or gynecology wards were not included in our study. The difference may also be explained by a potential overestimation of physical activity in our study. Although the standardized and world-widely used IPAQ-Short [26–28] was used to assess physical activity, with an average of 766 min of moderate physical activity per week, the reported physical activity appears unreasonably high. As overestimation of physical activity using the IPAQ-Short has also been observed in previous studies [34], the proportion of physically inactive patients and of patients with multiple BHRFs may be higher than identified in our study.

Our findings support previous findings on the co-occurrence of BHRFs in general hospital patients [10] while providing a more detailed assessment of unhealthy diet. It shows that more than half of the patients reported co-occurring BHRFs, when using overweight (53%), vegetable and fruit intake (62%) or the combined measure of 4 food components (66%) to measure unhealthy diet.

The combined measure of 4 food components identified 100% of the participants to not meet WHO recommendations for the intake of fat, fiber, salt and sugar. The much less time-consuming measures of vegetable/fruit intake and overweight also identified 87% and 62% of these participants, respectively. In particular with regard to vegetable and fruit intake, this may be considered acceptable sensitivity once the combined measure has been validated. The research on vegetable and fruit intake as diet indicator compared to other measures of diet is scarce. However, other studies that assessed multiple BHRFs mostly used overweight [6, 7, 9, 10] and vegetable and fruit intake [6, 8, 11] to measure unhealthy diet. The results of studies choosing more detailed measures based on dietary guidelines [35, 36] are limited in comparability due to different national guidelines and different aspects of diet included in the assessment. Overweight was disclosed by 64% of those whose diet was unhealthy according to vegetable and fruit intake. Vegetable and fruit intake has the advantage of measuring modifiable behavior and not a metabolic result. This enables the identification of people with an unhealthy diet regardless of overweight or obesity and reduces stigmatization of overweight people which might lead to higher compliance in interventions. Thus, vegetable and fruit intake seem to be a suitable diet indicator to help develop and implement behavioral interventions into routine care.

This study shows that the number of BHRFs is significantly associated with the number of self-reported sick days if any when using vegetable and fruit intake as indicator of unhealthy diet. This seems plausible as single BHRFs are associated with a higher amount of sick leave due to health disorders [17, 36]; and multiple BHRFs are associated with higher risk for chronic diseases [37]. However, although the effects were similar when using the other 2 indicators of unhealthy diet, the association between the number of BHRFs and sick days lacked significance. This may be due to lack of power given the small subgroup of patients who did report any sick days (*n* = 145).

### Strengths

Three strengths of this study should be noted. First, with 78% participation, the proactive recruitment strategy resulted in a satisfactory reach of hospital patients. Secondly, this study provided a more detailed assessment of diet than former studies investigating the co-occurrence of multiple BHRFs [7, 10, 37] by assessing the intake of the 4 single food components fat, fiber, salt and sugar and their combined measurement in addition to overweight and vegetable and fruit intake. Third, this study provides a detailed assessment of physical activity among general hospital patients, considering not only sport activity as measured earlier [10], but any type of phsysical activity as suggested by the WHO [38].

### Limitations

Several limitations of this study should be noted. Firstly, as the study was based on self-report, responses may be distorted by the tendency of people to provide socially desirable answers. Given that self-report is likely to lead to under-reporting as found for overweight [39] and as discussed above concerning the measurement of physical activity, proportions of BHRFs may be underestimated. To reduce bias, valid assessment instruments such as the AUDIT-C [18] were used whenever possible. Secondly, the assessment of fat, fiber, salt and sugar intake needs validation. Though the mean values concerning grams and kilocalories per day for each component obtained by the nutritional scientist appear to have some face validity, patients in our study had a higher fat intake and a lower intake of fiber, salt and sugar per day compared to the German general population [40]. Concerning fat and fiber, this may be plausible as it could be suspected that hospital patients may have unhealthier eating habits than the general population. The lower salt intake may be explained by an underestimation of salt intake in our measure as some food rich in salt and additional salt to season meals were not assessed. The unexpectedly low intake of sugar may be caused by under-reporting due to socially desirable answers as described above. Thirdly, the assessment of the physical activity level required for the determination of the individual recommended fat-intake per day did not include work-related physical activity. We tried to compensate for this by determining the physical activity level factor on the basis of patients’ self-reported moderate and vigorous physically active minutes per week. Fourthly, the small sample-size resulted in large confidence-intervals and uncertainty of reported proportions. Fifthly, the sample composition may be influenced by peculiarities of the covid-19 pandemic and related restrictions in hospital care. For example, compared to a previous study among patients with at-risk alcohol use [32], patients reported more sick days (28 vs. 13) 6 months prior to hospitalization. Unfortunately, the causes for the sick days were not assessed. Thus, we can only suspect that this large difference may be explained by longer waiting time for hospital treatment or by covid-19 related quarantine days in the past. Finally, this study does not allow cause-effect-statements.

### Conclusion

The findings of this study suggest that BHRFs are associated with the number of sick days. The majority of study participants had 2 or more BHRFs. Among the assessments of unhealthy diet, vegetable and fruit intake may be recommended if a time-saving estimate is needed.
